# Supplementary material for: Orthogonal Proteomic Platforms and Their Implications for the Stable Classification of High-Grade Serous Ovarian Cancer Subtypes
Source: iScience. 2020 Apr 18;23(6):101079. doi: 10.1016/j.isci.2020.101079 (PMC7298555; doi:10.1016/j.isci.2020.101079)
Supplement: Document S1. Transparent Methods and Figures S1–S5 [file mmc1.pdf]

**iScience, Volume *23***

## Supplemental Information

# Orthogonal Proteomic Platforms and Their Implications for the Stable Classification of High-Grade Serous Ovarian Cancer Subtypes

**Stefani N. Thomas, Betty Friedrich, Michael Schnaubelt, Daniel W. Chan, Hui Zhang, and Ruedi Aebersold**

## Supplemental Information

### Supplementary Figures

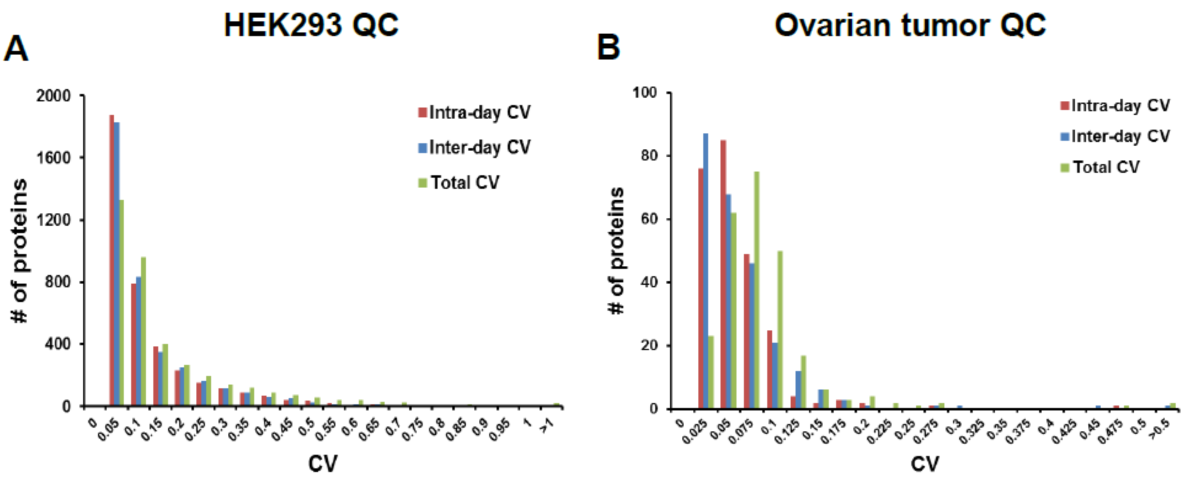


**Supplementary Figure S1, Related to Figure 2. Distribution of quantified proteins from QC strategies used to assess technical variability. a)** Distribution of quantified protein CVs from HEK293 lysate analyzed via SWATH (n=3 technical replicates per day for 3 days; total = 9 technical replicates) prior to the analysis of the ovarian tumors. Median total CV=15%; 3,855 quantified proteins. **b)** Distribution of quantified protein CVs from a control ovarian tumor analyzed in duplicate via DDA prior to and mid-way through the ovarian tumor SWATH analyses (n=4 technical replicates). Median total CV=7%; 781 quantified proteins.

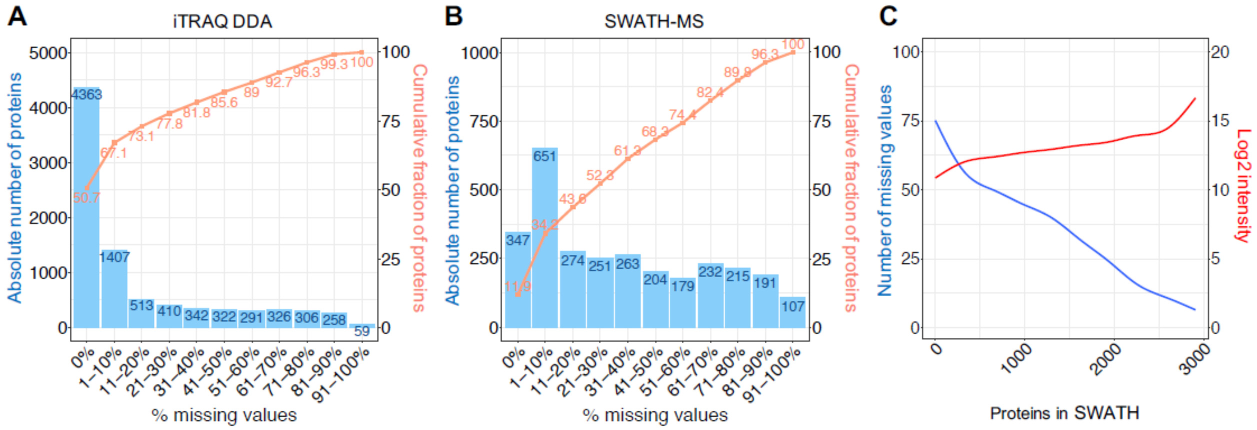


**Supplementary Figure S2, Related to Figure 2. Completeness of iTRAQ DDA and SWATH-MS proteomic data. a)** Analysis of missing values in the iTRAQ DDA dataset. **b)** Analysis of missing values in the SWATH-MS dataset. **c)** Relationship between signal intensity and number of missing values for the proteins quantified by SWATH-MS.

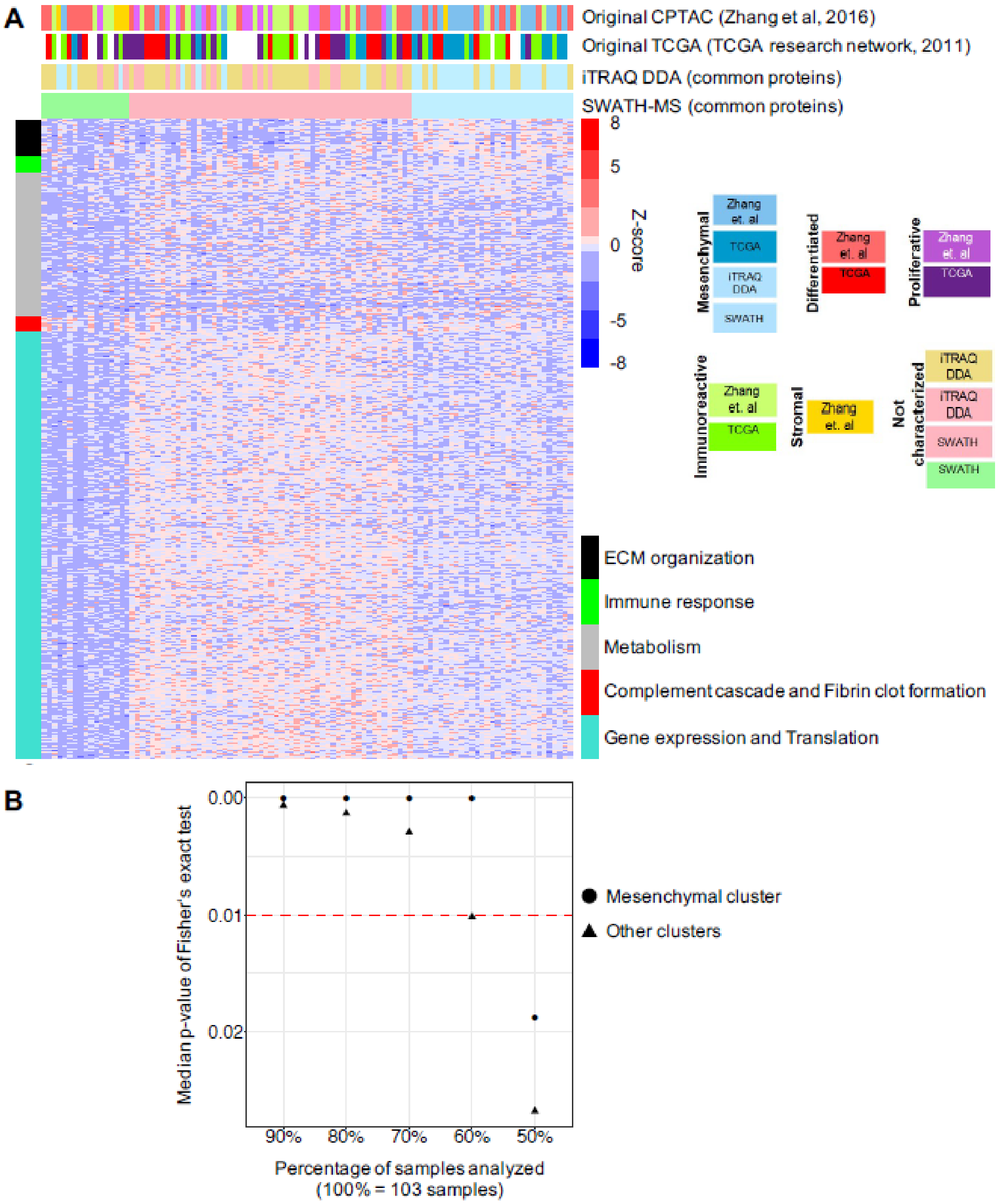


**Supplementary Figure S3, Related to Figure 3. Protein (iTRAQ DDA, SWATH-MS) and mRNA-based high-grade serous ovarian cancer subtype classification. a)** Tumor subtype classification based on the associated driving protein modules. Numbers indicate z-scores. Subtype classification: Blue – Mesenchymal; Red – Differentiated; Purple – Proliferative; Green – Immunoreactive; Yellow – Stromal. **b)** Stability of the Mesenchymal compared to the non-Mesenchymal subtypes assessed by bootstrapping and Fisher's exact test.

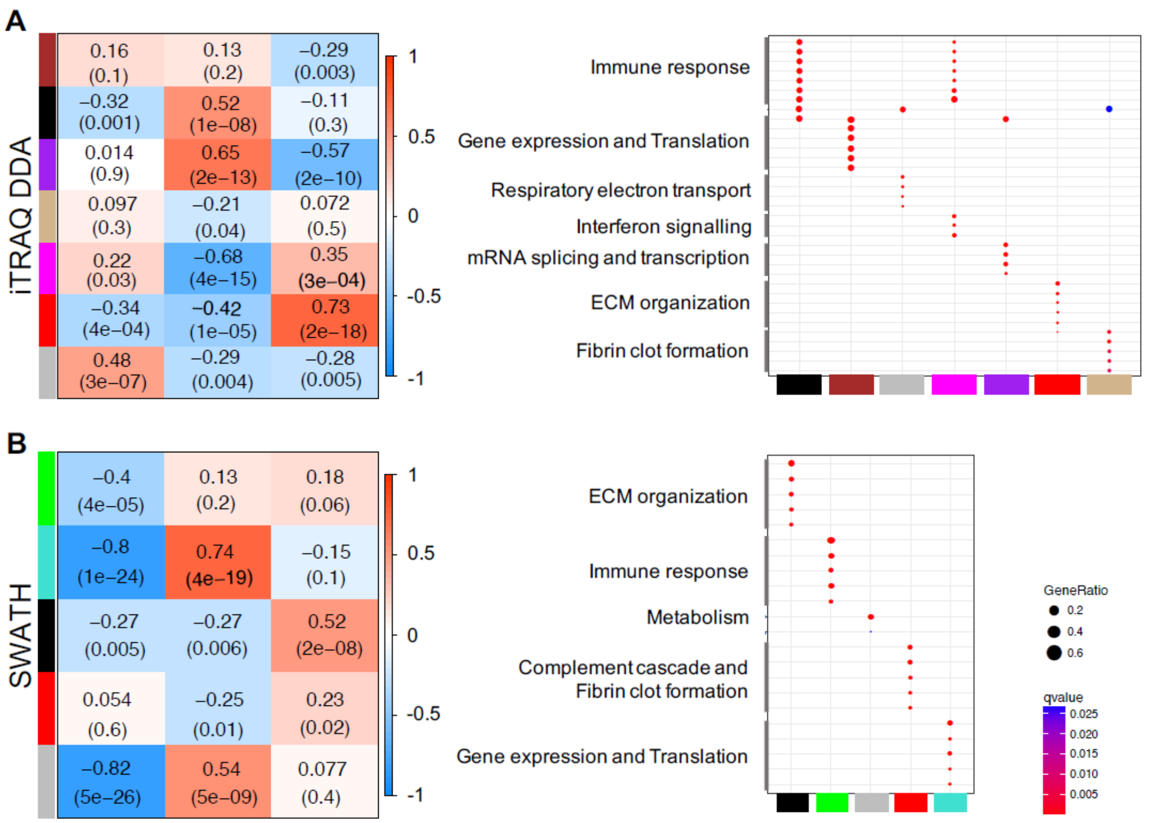


**Supplementary Figure S4, Related to Figure 3. Analysis of tumor subtype analysis according to data type.** Correlation of weighted gene co-expression network analysis (WGCNA)-derived protein modules with proteomic subtypes derived from the iTRAQ DDA data (**a**) and the SWATH-MS data (**b**). The numbers in each box indicate the Pearson correlation coefficient (-1 to 1), and the numbers in parentheses indicate the p-value corresponding to the significance of each association. The enrichment of Reactome pathways in the WGCNA-derived modules in the iTRAQ DDA and SWATH-MS data is also shown. The significance of association is denoted by colored dots indicating the q-value, and the number of proteins mapped to a given ontology as a proportion of the total number of proteins in the module (GeneRatio) is indicated by the size of the dot. The WGCNA-derived protein modules for the iTRAQ DDA data are as follows: magenta – gene expression; black – immune response; purple – transcription; tan – erythrocyte and platelet; magenta – cytokine signaling; red – ECM interaction; gray – metabolism. The WGCNA-derived protein modules for the SWATH data are as follows: green – immune response; turquoise – gene expression; black – ECM interaction; red – complement cascade; gray – metabolism.

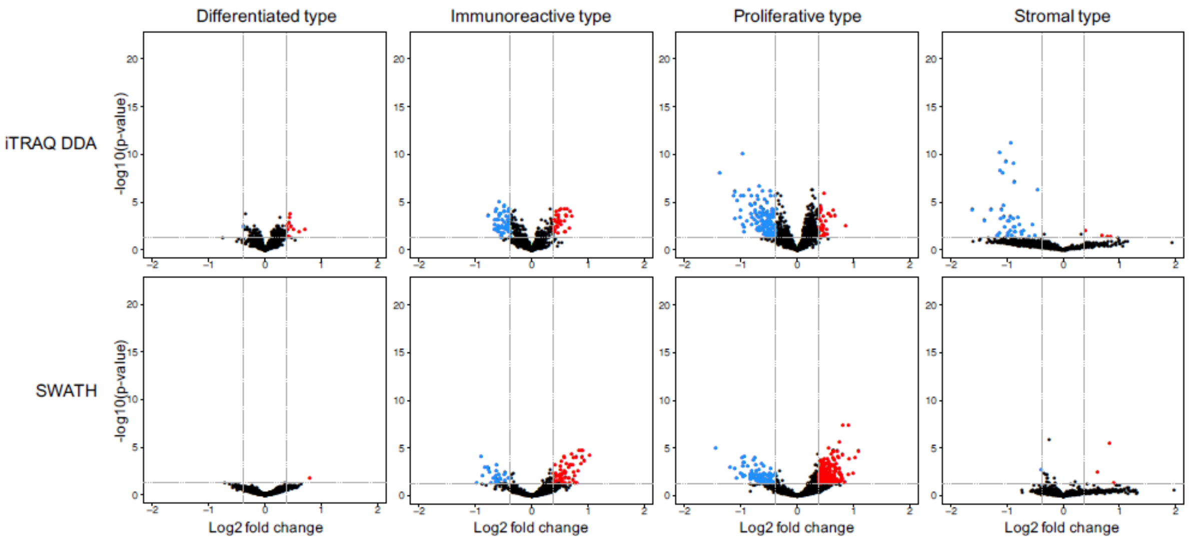


**Supplementary Figure S5, Related to Figure 3. Differential relative protein abundance analysis of proteins in the Differentiated, Immunoreactive, Proliferative and Stromal subtype tumors from the iTRAQ DDA and SWATH-MS datasets.** Up-regulated proteins are indicated in red and the down-regulated proteins are indicated in blue. The $log_2$ fold-change cut-off was 1.3 and the significance cut-off was set at $p<0.05$.

# Transparent Methods

# STAR Methods

## KEY RESOURCES TABLE

| REAGENT or RESOURCE | SOURCE | IDENTIFIER |
|---|---|---|
| **Biological Samples** | | |
| Human ovarian tumor tissue | National Cancer Institute Clinical Proteomic Tumor Analysis Consortium | https://proteomics.cancer.gov/programs/cptac |
| **Chemicals, Peptides, and Recombinant Proteins** | | |
| Urea | Sigma-Aldrich | Cat# U0631 |
| HPLC-grade water | J.T. Baker | Cat# 4218-03 |
| Triethylammonium bicarbonate | Sigma-Aldrich | Cat# T7408 |
| Ammonium bicarbonate | Sigma-Aldrich | Cat# A6141 |
| Tris (2-carboxyethyl) phosphine (TCEP) | Thermo Fisher Scientific | Cat# 20490 |
| Iodoacetamide | Sigma-Aldrich | Cat# A3221 |
| Sequencing-grade modified trypsin | Promega | Cat# V511X |
| Formic acid | Sigma-Aldrich | Cat# 56302 |
| Trifluoroacetic acid | Sigma-Aldrich | Cat# 91707 |
| Acetonitrile | J.T. Baker | Cat# 9829-03 |
| iTRAQ reagent kit – 4plex | Sciex | Cat# 4352135 |
| **Critical Commercial Assays** | | |
| BCA bicinchoninic protein assay kit | Pierce | Cat# 23325 |
| **Deposited Data** | | |
| iTRAQ DDA data | NCI CPTAC Data Portal | https://cptac-data-portal.georgetown.edu/cptac/s/S026 (Mass spectrometry site: Johns Hopkins University) |
| SWATH data, input spectral library, OpenSWATH results | ProteomeXchange Consortium | http://proteomecentral.proteomexchange.org Dataset identifier: PXD010437 |
| **Software and Algorithms** | | |
| Programming code for bioinformatics pipeline | Github repository | bfriedrichgrube/OC_CPTAC_iTRAQ_SWATH |
| msconvert | ProteoWizard v3.0.8851 | http://proteowizard.sourceforge.net/download.html |
| X!Tandem 2013.06.25.1 | Duncan et al., 2005 | https://www.thegpm.org/tandem/ |

| | | |
|---|---|---|
| OMSSA 2.1.9 | Geer et al., 2004 | https://omictools.com/omssa-tool |
| Comet 2015.02 rev. 3 | Eng et al., 2013 | http://comet-ms.sourceforge.net/ |
| Trans-Proteomic Pipeline v. 4.7 | Keller et al., 2005; Lam et al., 2007 | http://tools.proteomecenter.org/wiki/index.php?title=Software:TPP |
| OpenSWATH | Rost et al., 2014 | http://www.openswath.org/en/latest/ |
| SpectraST | Keller et al., 2005; Lam et al., 2007 | http://tools.proteomecenter.org/wiki/index.php?title=SpectraST |
| TRIC | Rost et al., 2016 | http://proteomics.ethz.ch/tric/ |
| mapDIA v. 3.0.2 | Teo et al., 2015 | http://mapdia.sourceforge.net/Main.html |
| **Other** | | |
| SepPak tC18 Vac cartridges | Waters | Cat# WAT054925 |
| Polysulfoethyl A strong cation exchange columns | Glygen | Cat# TT3SSA |

## CONTACT FOR REAGENT AND RESOURCE SHARING

Further information and requests for resources and reagents should be directed to and will be fulfilled by the Lead Contact, Hui Zhang (huizhang@jhu.edu).

## EXPERIMENTAL MODEL AND SUBJECT DETAILS

**Tumor samples** The tumor specimens were obtained through The Cancer Genome Atlas (TCGA) Biospecimen Core Resource, and they were previously genomically characterized (Cancer Genome Atlas Research, 2011). As previously described (Zhang et al., 2016), the biospecimens were obtained from newly diagnosed patients with ovarian serous adenocarcinoma who were undergoing surgical resection and did not receive prior treatment, including chemotherapy or radiotherapy, for their disease. Per the TCGA study, all specimens were obtained from patients with appropriate consent from the relevant institutional review board. Frozen tissue specimens were extracted and used for subsequent proteomic analysis.

## METHOD DETAILS

**Protein extraction and in-solution digestion** Approximately 50mg of each tumor tissue specimen was sonicated in 1.5mL of 8M urea, 0.8M $NH_4HCO_3$, pH 8.0. Protein concentration was determined using a BCA assay (Thermo Fisher Scientific). Protein disulfide bonds were reduced with 10mM tris (2-carboxyethyl) phosphine (TCEP) for 1h at 37°C, followed by alkylation with 12mM iodoacetamide for 1h at RT in the dark. After dilution 1:4 with deionized water, proteins were digested with sequencing-grade modified trypsin (Promega, Madison, WI) (1:50 enzyme:protein, weight/weight) for 12h at 37°C. This was followed by the addition of an aliquot of the same amount of trypsin and incubation overnight at 37°C. The digested samples were acidified with 10% trifluoroacetic acid (TFA) to pH<3, de-salted using strong cation exchange and C18 solid-phase extraction (SPE) columns (Waters, Milford, MA) and dried using a Speed-Vac.

**Shotgun proteomics using iTRAQ data-dependent acquisition (DDA)** Relative quantitative proteomic analysis was conducted using 4-plex isobaric tags for relative and absolute quantitation (iTRAQ) reagents (Sciex) as previously described (Zhang et al., 2016). Peptides (500μg) were dissolved in 150μL of 0.5M triethylammonium bicarbonate, pH 8.5 and combined with 5U of 4-plex iTRAQ reagent dissolved in ethanol followed by a 2h incubation at RT, quenching with 10% TFA, and de-salting using C18 SPE columns. iTRAQ channel 114 was used to label the reference sample which was created by pooling an aliquot from each individual tumor sample. Offline basic reversed phase liquid chromatography (bRPLC) was conducted using a Zorbax extend 4.6 x 100mm C-18 column (Agilent) and an Agilent 1220 Infinity HPLC system to reduce the sample complexity prior to mass spectrometry analysis. A total of 96 fractions were collected and concatenated into 24 fractions. The fractions were dried in a Speed-Vac and stored at -80°C until analysis by LC-MS/MS using an LTQ Orbitrap Velos mass spectrometer (Thermo Scientific). Peptides were loaded onto a 2cm guard column (Thermo Scientific) and separated on a 75μm x 15cm Acclaim PepMap100 column (Thermo Scientific) using a Dionex Ultimate 3000 RSLC nano chromatography system (Thermo Scientific). The LC gradient profile was 2-22% B for 70min, 22-29% B for 8min, 29-95% B for 4min, and 95% B for 8min, where mobile phase B was 0.1% formic acid in acetonitrile, and mobile phase

A was 0.1% formic acid in water. Orbitrap full MS spectra were collected from 400-1800 m/z at a resolution of 30,000 followed by data-dependent MS/MS (7,500 resolution) of the ten most abundant ions. Charge-state screening was enabled to prevent the acquisition of MS/MS spectra for ions with unassigned or single charges. Dynamic exclusion (40s duration) was enabled to minimize the repeated acquisition of previously acquired MS/MS spectra.

**Proteomic analysis using SWATH mass spectrometry** SWATH-MS measurements were conducted at the Johns Hopkins University using a Sciex 5600$^+$ TripleTOF mass spectrometer interfaced with an Eksigent ekspert nanoLC 425 cHiPLC system. Peptides (1µg) were loaded onto a 6mm x 200µm ChromXP C18-CL 3µm, 120Å trap column followed by separation on a 75µm x 15cm ChromXP C18-CL 3µm, 120Å Nano cHiPLC column using a 120min method (90min gradient from 3-35% B – 0.1% formic acid in acetonitrile) at a flow rate of 300 nL/min. To create the spectral library for the SWATH-MS data analysis, each sample was run individually (1µg peptides per injection) using a data-dependent data acquisition (DDA) method wherein MS spectra were acquired across a range of 400-1800 m/z followed by the acquisition of MS/MS spectra of the top 30 most intense precursor ions with a charge state of z=2-5. The spectral library was also comprised of mass spectrometry data acquired from a fractionated (48 fractions) pool of peptides from all 103 tumors. Each of the 48 fractions from the pooled sample was analyzed using the same DDA method described above. SWATH data of the individual tumors were acquired using a variable window strategy wherein the sizes of the precursor ion selection windows were inversely related to m/z density. The average window width for precursor ion selection was 12 m/z with a range of 6-25 m/z. The collision energy was optimized for each window according to the calculation for a charge 2+ ion centered in the window with a spread of 5 eV. The MS accumulation time was 250ms and the MS/MS accumulation time for fragment ions accumulated in high sensitivity mode was 50ms, resulting in a total duty cycle of approximately 3.5s. To assess the analytical precision of the proteomics measurements, peptides from trypsin-digested HEK293 cell proteins were analyzed via SWATH-MS in triplicate immediately prior to the SWATH-MS analysis of the 103 individual tumor samples. Additionally, an ovarian cancer tumor sample separate

from the TCGA collection was used as a QC sample. This specimen was analyzed using the DDA method described above in duplicate prior to the SWATH-MS analysis of the 103 individual tumor samples and 10 days later in duplicate mid-way through the analysis of the individual tumor samples. Instrument performance was assessed daily by monitoring the peak area of 5 peptides from a trypsin-digested *E. coli* ß-Galactosidase LC-MS standard that was injected every day a sample was run on the mass spectrometer.

**iTRAQ DDA data processing** iTRAQ DDA data from the Johns Hopkins CPTAC center was analyzed by the Common Data Analysis Pipeline (CDAP) and downloaded from the NCI CPTAC Data Portal (https://cptac-data-portal.georgetown.edu/cptac/s/S026); mass spectrometry site: Johns Hopkins University (Edwards et al., 2015; Ellis et al., 2013). Values obtained from proteotypic peptides were chosen for further analysis. The protein matrix contained 8597 proteins. The protein matrix was filtered for proteins without missing values (4363 proteins). PSM files were additionally downloaded from the CPTAC Data Portal and processed following the CDAP to the peptide level data in order to investigate peptide variability.

**SWATH-MS data processing** Raw mass spectrometry measurements obtained from the TripleTOF 5600$^+$ in DDA and SWATH mode were converted to mzXML file format using msconvert (ProteoWizard v3.0.8851) (Chambers et al., 2012). DDA measurements from all 103 samples and 48 fractions of a pooled sample were searched with X!Tandem (2013.06.25.1) (Duncan et al., 2005), OMSSA (2.1.9) (Geer et al., 2004) and Comet (2015.02 rev. 3) (Eng et al., 2013). Identified peptides were processed through the Trans-Proteomic Pipeline (TPP v.4.7 Polar Vortex rev 0) using PeptideProphet, iProphet and ProteinProphet scoring (FDR <0.01) and SpectraST (Keller et al., 2005; Lam et al., 2007). The assay library was built according to (Schubert et al., 2015) using a set of 113 common internal retention time standards (ciRTs) (Parker et al., 2015). The resulting library contained 8897 proteins supported by proteotypic peptides.

SWATH data was analyzed using OpenSWATH (Rost et al., 2014) from OpenMS (v.1.10.0) with the previously described sample-specific assay library. FDR was controlled

using PyProphet (v.0.0.19) allowing for a peptide-query FDR of 1% and protein-FDR of 1% as described by Rosenberger et al. (Rosenberger et al., 2017). Runs were aligned for improved quantification using TRIC (msproteomicstools master branch from GitHub commit c10a2b8) (Rost et al., 2016).

OpenSWATH results were annotated and formatted using the R package SWATH2stats (v.1.0.3) (Blattmann et al., 2016) prior to quantile normalization (preprocessCore v.1.32.0) (Bolstad, 2017) and batch-wise mean-centering batch correction of known sample preparation batches. Fragment-level data was then fed into mapDIA (v.3.0.2) (Teo et al., 2015) in order to filter for outliers and proteins with at least 3 fragments per peptide and 2 peptides per protein. The 3 most intense fragments per peptide were summed and the resulting peptide intensities were divided by the mean peptide intensity (as an internal reference) over all runs to obtain a similar data structure as in iTRAQ DDA. Peptide ratios were averaged per protein. A total of 2914 proteins were quantified in this manner across all 103 samples.

An additional filtering and imputation step was conducted to mitigate the missing values in the SWATH data. **Figure S2B** shows the number of missing values in the SWATH data and their occurrence dependent on the protein abundance represented by $log_2$ intensity (**Figure S2C**). As expected, there is an inverse relationship between protein abundance and missing values. Missing values in low abundance proteins are more likely to occur due to technical reasons, while missing values amongst high abundance proteins potentially have biological significance which might be important for further analysis. We therefore filtered the protein matrix based on the occurrence of abundance-dependent missing values. We confined the number of missing values using a stricter threshold for low abundance proteins than high abundance proteins according to the following rules: no missing values were allowed for the lowest abundant 10% of proteins, 10% missing values were allowed for the next 10% abundant proteins and so on until 90% missing values were allowed for the highest abundant proteins. A total of 1659 proteins remained that passed these filtering steps. The missing values from those proteins were filled using an imputation method based on what is used by Perseus software (Tyanova et al., 2016)

wherein random numbers forming a normal distribution were generated fitting in the left end of the distribution of the measured values, representing values within the noise.

## QUANTIFICATION AND STATISTICAL ANALYSIS

### Bioinformatics pipeline

Bioinformatic analyses were performed in R (v.3.2.2 “Fire Safety”).

*Technical evaluation.* A total of 1599 proteins common to the iTRAQ DDA and SWATH data sets were evaluated for their correlation and variability (**Figure 2**). The distribution of values in both data sets was plotted using the R package vioplot (v.0.2) (Adler, 2005). Spearman’s rank correlation was computed for each protein across all 103 samples in both data sets. The resulting Spearman’s rho values were ordered and the median was calculated. For peptide variability, a CV-like score was calculated for the peptides from each protein (standard deviation of peptides per protein divided by mean peptide intensity per protein). The distribution of the resulting CVs was plotted with vioplot.

*Molecular classification comparison.* Protein matrices were transformed into z-scores and a molecular classification was obtained by running a model-based clustering with mclust from the R package mclust under default settings (v.5.2) (Fraley and Raftery, 2002; Fraley et al., 2012). Protein modules characterizing sample groups were obtained with weighted gene-correlation network analysis (WGCNA v.1.51) (Langfelder and Horvath, 2008, 2012) and functional enrichment with clusterProfiler (v.2.4.3) (Yu et al., 2012) (**Figure S4**). The resulting molecular classifications were compared based on their similarity using the Adjusted Rand Index (ARI), a measure for classification similarity, implemented within the R package mclust previously used. The heat map displayed in **Figure S3A** was generated based on SWATH z-scores using pheatmap (v.1.0.8) (Kolde, 2015). The stability of the classification results was evaluated by bootstrapping. Different fractions of samples or proteins were drawn 100 times and classified using mclust with default settings, and the similarity to the molecular classification of the full data set was compared with the ARI (**Figure 4**).

In order to assess the stability of the mesenchymal class a Fisher's exact test was performed on the mesenchymal class and the other resulting classes with a bootstrapping approach by drawing a fraction of samples 100 times (**Figure S3B**). Group comparison of the original classes from CPTAC (Zhang et al., 2016) were obtained by a student's t-test (**Figure 6** and **S4**).

*HRD group comparison.* Group comparison to identify the differentially expressed proteins in the HRD vs. non-HRD patients was conducted using mapDIA (v.3.0.2) (Teo et al., 2015) on fragment ion-level data (**Figure 7A**). The resulting fold changes were used as input signal in a network propagation approach (Hofree et al., 2013) called Network Smoothing (R package BioNetSmooth v.1.0.0) (Chokkalingam et al., 2016).

As an input network the STRING database for human (Taxon 9606 v.10.5) was downloaded and filtered for interactions with experimental evidence and a score >800. The top 5% of negative and positive scores were used for further investigation of functional enrichment of Gene Ontology using the STRING database (STRING-db.org v.10.5 accessed on 2018/03/14).

## DATA AND SOFTWARE AVAILABILITY

### Mass spectrometry data

All the raw data from SWATH-MS measurements, along with the input spectral library and OpenSWATH results can be freely downloaded from the ProteomeXchange Consortium (http://proteomecentral.proteomexchange.org) with the dataset identifier: PXD010437 via the PRIDE partner (Vizcaino et al., 2016).

Reviewer account details:

Username: reviewer95375@ebi.ac.uk

Password: 9VoPWx2g

The iTRAQ DDA data are available on the NCI CPTAC Data Portal (https://cptac-data-portal.georgetown.edu/cptac/s/S026); mass spectrometry site: Johns Hopkins University. Other data are available from the corresponding authors upon request.

## Programming codes

All code used for the downstream bioinformatics pipeline can be obtained from the github repository (bfriedrichgrube/OC_CPTAC_iTRAQ_SWATH).

## SUPPLEMENTARY REFERENCES

Adler, D. (2005). vioplot: Violin plot. R package version 0.2.

Blattmann, P., Heusel, M., and Aebersold, R. (2016). SWATH2stats: An R/Bioconductor Package to Process and Convert Quantitative SWATH-MS Proteomics Data for Downstream Analysis Tools. PLoS One *11*, e0153160.

Bolstad, B. (2017). preprocessCore: A collection of pre-processing functions. R package version 1.32.0.

Cancer Genome Atlas Research, N. (2011). Integrated genomic analyses of ovarian carcinoma. Nature *474*, 609-615.

Chambers, M.C., Maclean, B., Burke, R., Amodei, D., Ruderman, D.L., Neumann, S., Gatto, L., Fischer, B., Pratt, B., Egertson, J., *et al.* (2012). A cross-platform toolkit for mass spectrometry and proteomics. Nat Biotechnol *30*, 918-920.

Chokkalingam, M., Debes, C., and Beyer, a. (2016). BioNetSmooth: Spreads signal on interaction network to identify altered subnetworks. R package v. 1.0.0.

Duncan, D.T., Craig, R., and Link, A.J. (2005). Parallel tandem: a program for parallel processing of tandem mass spectra using PVM or MPI and X!Tandem. J Proteome Res *4*, 1842-1847.

Edwards, N.J., Oberti, M., Thangudu, R.R., Cai, S., McGarvey, P.B., Jacob, S., Madhavan, S., and Ketchum, K.A. (2015). The CPTAC Data Portal: A Resource for Cancer Proteomics Research. J Proteome Res *14*, 2707-2713.

Ellis, M.J., Gillette, M., Carr, S.A., Paulovich, A.G., Smith, R.D., Rodland, K.K., Townsend, R.R., Kinsinger, C., Mesri, M., Rodriguez, H., *et al.* (2013). Connecting genomic alterations to cancer biology with proteomics: the NCI Clinical Proteomic Tumor Analysis Consortium. Cancer Discov *3*, 1108-1112.

Eng, J.K., Jahan, T.A., and Hoopmann, M.R. (2013). Comet: an open-source MS/MS sequence database search tool. Proteomics *13*, 22-24.

Fraley, C., and Raftery, A. (2002). Model-based clustering, discriminant analysis, and density estimation. Journal of the American Statistical Association *97*, 611-631.

Fraley, C., Raftery, A., Murphy, T., and Scrucca, L. (2012). mclust Version 4 for R: Normal Mixture Modeling for Model-Based Clustering, Classification, and Density Estimation University of Washington Department of Statistics Technical Report No 597.

Geer, L.Y., Markey, S.P., Kowalak, J.A., Wagner, L., Xu, M., Maynard, D.M., Yang, X., Shi, W., and Bryant, S.H. (2004). Open mass spectrometry search algorithm. J Proteome Res *3*, 958-964.

Hofree, M., Shen, J.P., Carter, H., Gross, A., and Ideker, T. (2013). Network-based stratification of tumor mutations. Nat Methods *10*, 1108-1115.

Keller, A., Eng, J., Zhang, N., Li, X.J., and Aebersold, R. (2005). A uniform proteomics MS/MS analysis platform utilizing open XML file formats. Mol Syst Biol *1*, 2005 0017.

Kolde, R. (2015). pheatmap: Pretty Heatmaps. R package version 1.0.8.

Lam, H., Deutsch, E.W., Eddes, J.S., Eng, J.K., King, N., Stein, S.E., and Aebersold, R. (2007). Development and validation of a spectral library searching method for peptide identification from MS/MS. Proteomics *7*, 655-667.

Langfelder, P., and Horvath, S. (2008). WGCNA: an R package for weighted correlation network analysis. BMC Bioinformatics *9*, 559.

Langfelder, P., and Horvath, S. (2012). Fast R Functions for Robust Correlations and Hierarchical Clustering. J Stat Softw *46*.

Parker, S.J., Rost, H., Rosenberger, G., Collins, B.C., Malmstrom, L., Amodei, D., Venkatraman, V., Raedschelders, K., Van Eyk, J.E., and Aebersold, R. (2015). Identification of a Set of Conserved Eukaryotic Internal Retention Time Standards for Data-independent Acquisition Mass Spectrometry. Mol Cell Proteomics *14*, 2800-2813.

Rosenberger, G., Bludau, I., Schmitt, U., Heusel, M., Hunter, C.L., Liu, Y., MacCoss, M.J., MacLean, B.X., Nesvizhskii, A.I., Pedrioli, P.G.A.*, et al.* (2017). Statistical control of peptide and protein error rates in large-scale targeted data-independent acquisition analyses. Nat Methods *14*, 921-927.

Rost, H.L., Liu, Y., D'Agostino, G., Zanella, M., Navarro, P., Rosenberger, G., Collins, B.C., Gillet, L., Testa, G., Malmstrom, L.*, et al.* (2016). TRIC: an automated alignment strategy for reproducible protein quantification in targeted proteomics. Nat Methods *13*, 777-783.

Rost, H.L., Rosenberger, G., Navarro, P., Gillet, L., Miladinovic, S.M., Schubert, O.T., Wolski, W., Collins, B.C., Malmstrom, J., Malmstrom, L.*, et al.* (2014). OpenSWATH enables automated, targeted analysis of data-independent acquisition MS data. Nat Biotechnol *32*, 219-223.

Schubert, O.T., Gillet, L.C., Collins, B.C., Navarro, P., Rosenberger, G., Wolski, W.E., Lam, H., Amodei, D., Mallick, P., MacLean, B.*, et al.* (2015). Building high-quality assay libraries for targeted analysis of SWATH MS data. Nat Protoc *10*, 426-441.

Teo, G., Kim, S., Tsou, C.C., Collins, B., Gingras, A.C., Nesvizhskii, A.I., and Choi, H. (2015). mapDIA: Preprocessing and statistical analysis of quantitative proteomics data from data independent acquisition mass spectrometry. J Proteomics *129*, 108-120.

Tyanova, S., Temu, T., Sinitcyn, P., Carlson, A., Hein, M.Y., Geiger, T., Mann, M., and Cox, J. (2016). The Perseus computational platform for comprehensive analysis of (prote)omics data. Nat Methods *13*, 731-740.

Vizcaino, J.A., Csordas, A., del-Toro, N., Dianes, J.A., Griss, J., Lavidas, I., Mayer, G., Perez-Riverol, Y., Reisinger, F., Ternent, T., *et al.* (2016). 2016 update of the PRIDE database and its related tools. Nucleic Acids Res *44*, D447-456.

Yu, G., Wang, L.G., Han, Y., and He, Q.Y. (2012). clusterProfiler: an R package for comparing biological themes among gene clusters. OMICS *16*, 284-287.

Zhang, H., Liu, T., Zhang, Z., Payne, S.H., Zhang, B., McDermott, J.E., Zhou, J.Y., Petyuk, V.A., Chen, L., Ray, D., *et al.* (2016). Integrated Proteogenomic Characterization of Human High-Grade Serous Ovarian Cancer. Cell.
